# Crystal structure of 2-{[1-(2-methyl-5-nitro-1*H*-imidazol-1-yl)propan-2-yl­oxy]carbon­yl}benzoic acid

**DOI:** 10.1107/S1600536814023927

**Published:** 2014-11-05

**Authors:** Hafiz Abdullah Shahid, Sajid Jahangir, Syed Adnan Ali Shah, Hamizah Mohd Zaki, Humera Naz

**Affiliations:** a Department of Chemistry, Faculty of Science, Federal Urdu University of Arts, Science and Technology, Gulshan-e-Iqbal, Karachi 75300, Pakistan; b Atta-ur-Rahman Institute for Natural Product Discovery, Universiti Teknologi MARA (UiTM), Puncak Alam Campus, 42300 Bandar Puncak Alam, Selangor D. E., Malaysia; c Faculty of Pharmacy, Universiti Tecknologi MARA, Puncak Alam, 42300 Selangor, Malaysia; d Faculty of Applied Sciences, Universiti Teknologi MARA (UiTM), 40450 Shah Alam, Malaysia

**Keywords:** crystal structure, nitro­imidazoles, O—H⋯N hydrogen bonds, pharmaceuticals

## Abstract

In the title compound, C_15_H_15_N_3_O_6_, the dihedral angle between the planes of the benzene and imidazole rings is 34.93 (10)°. An intra­molecular C—H⋯O hydrogen bond is observed. In the crystal, O—H⋯N hydrogen bonds link the mol­ecules into chains parallel to the *c* axis.

## Related literature   

For the applications and biological activities of nitro­imidazole and its derivatives, see: Maeda *et al.* (1953 ▶); Larina & Lopyrev (2009 ▶); Zhang *et al.* (2014 ▶); Gillis & Wiseman (1996 ▶). For the crystal structure of related compounds, see: Xiao *et al.* (2008 ▶); Shahid *et al.* (2014 ▶).
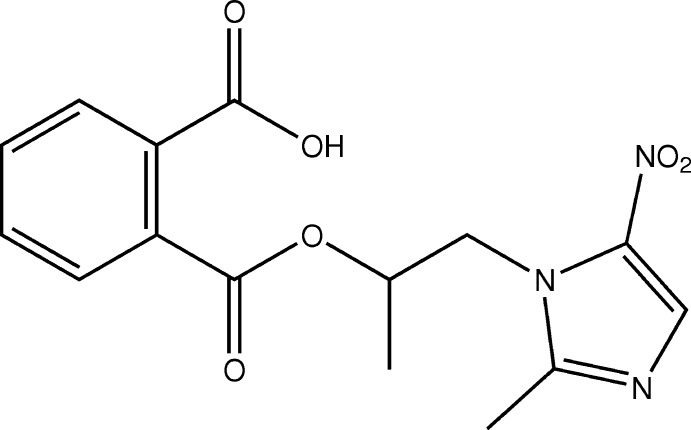


## Experimental   

### Crystal data  

C_15_H_15_N_3_O_6_
*M* *_r_* = 333.30
Monoclinic, 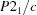

*a* = 11.189 (3) Å
*b* = 6.9489 (17) Å
*c* = 19.979 (5) Å
β = 98.056 (10)°
*V* = 1538.0 (6) Å^3^
*Z* = 4
Mo *K*α radiation
μ = 0.11 mm^−1^
*T* = 296 K
0.50 × 0.50 × 0.38 mm

### Data collection  

Bruker SMART APEX CCD area-detector diffractometer
Absorption correction: multi-scan (*SADABS*; Bruker, 2000 ▶) *T*_min_ = 0.946, *T*_max_ = 0.958
20047 measured reflections
2865 independent reflections
2515 reflections with *I* > 2σ(*I*)
*R*_int_ = 0.082

### Refinement  

*R*[*F* ^2^ > 2σ(*F* ^2^)] = 0.047
*wR*(*F* ^2^) = 0.119
*S* = 1.07
2865 reflections
222 parameters
H atoms treated by a mixture of independent and constrained refinement
Δρ_max_ = 0.31 e Å^−3^
Δρ_min_ = −0.22 e Å^−3^

### 

Data collection: *SMART* (Bruker, 2000 ▶); cell refinement: *SAINT* (Bruker, 2000 ▶); data reduction: *SAINT*; program(s) used to solve structure: *SHELXTL* (Sheldrick 2008 ▶); program(s) used to refine structure: *SHELXL2013* (Sheldrick, 2008 ▶); molecular graphics: *SHELXTL*; software used to prepare material for publication: *SHELXTL*, *PARST* (Nardelli, 1995 ▶) and *PLATON* (Spek, 2009 ▶).

## Supplementary Material

Crystal structure: contains datablock(s) global, I. DOI: 10.1107/S1600536814023927/rz5138sup1.cif

Structure factors: contains datablock(s) I. DOI: 10.1107/S1600536814023927/rz5138Isup2.hkl

Supporting information file. DOI: 10.1107/S1600536814023927/rz5138Isup3.cml

**Fig. 1.**
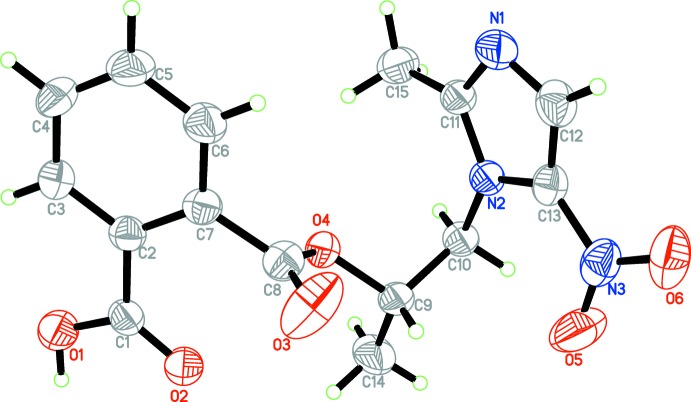
The molecular structure of the title compound with displacement ellipsoids drawn at 50% probability level.

**Fig. 2.**
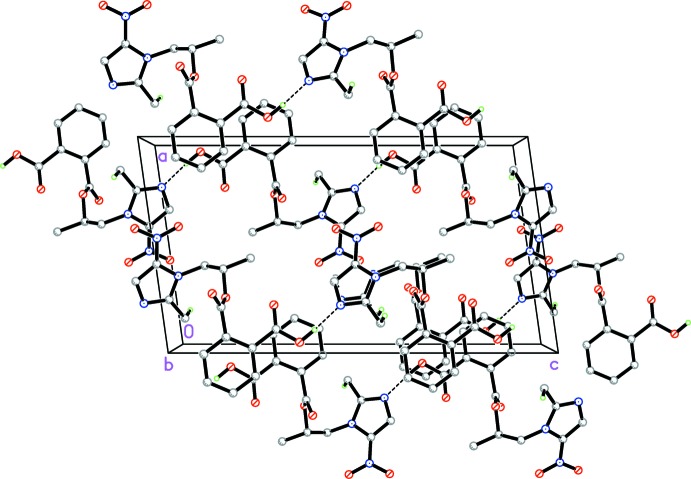
Crystal packing of the title compound viewed down the b axis. Only hydrogen atoms involved in O—H···N hydrogen bonds (dashed lines) are shown.

. DOI: 10.1107/S1600536814023927/rz5138fig1.tif
The mol­ecular structure of the title compound with displacement ellipsoids drawn at 50% probability level.

b . DOI: 10.1107/S1600536814023927/rz5138fig2.tif
Crystal packing of the title compound viewed down the *b* axis. Only hydrogen atoms involved in O—H⋯N hydrogen bonds (dashed lines) are shown.

CCDC reference: 1031694

Additional supporting information:  crystallographic information; 3D view; checkCIF report

## Figures and Tables

**Table 1:** Hydrogen-bond geometry (, )

*D*H*A*	*D*H	H*A*	*D* *A*	*D*H*A*
C9H9*A*O5	0.98	2.53	3.115(3)	118
O1H1*D*N1^i^	0.96(3)	1.78(3)	2.730(2)	175(3)

Symmetry code: (i) 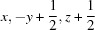.

## supplementary crystallographic information

### S1. Comment

Nitroimidazoles are well recognized as antibacterial agents since the early 1950s as azomycin, i.e. 2-nitroimidazole, was discovered (Maeda *et al.*, 1953). The imidazole moiety has a wide range of biological activities such as anticancer, antifungal, antibacterial, antitubercular, antiparasitic, antihistaminic, antineuropathic, antihypertensive, anti-inflammatory, antiobesity, antiviral, antitumor, antihelmintic, antiallergic, antineoplastic, local analgesic, and spazmolytic activities (Larina & Lopyrev, 2009; Zhang *et al.*, 2014). Nowadays, various drugs are available which belongs to the nitroimidazole class such as secnidazole (Flagentyl), metronidazole (Flagyl), ornidazole (Xynor), tinidazole (Fasigyn) and others. Secnidazole is an efficient drug in the treatment of protozoal infections. Secnidazole has been explored for the treatment of amoebiasis, giardiasis, urogenital trichomoniasis and nonspecific bacterial vaginosis (Gillis & Wiseman, 1996).

In the title compound (Fig. 1), the mean planes through the benzene (C2–C7) and imidazole (N2/C11/N1/C12/C13) rings form a dihedral angle of 34.93 (10)°. The bond lengths and angles are within the normal ranges and in agreement with those observed in related compounds (Xiao *et al.*, 2008; Shahid *et al.*; 2014). The molecular conformation is stabilized by a non-classical C—H···O hydrogen bond (Table 1). In the crystal, molecules are linked into chains parallel to the *c* axis *via* intermolecular O—H···N hydrogen bonds (Table 1, Fig. 2).

### S2. Experimental

For the synthesis of title compound, 10.8 mmol of secnidazole (2 g) and 10.8 mmol of phthalic anhydride (1.6 g) were dissolved in a mixture of acetone (15 ml) and pyridine (1 ml). The reaction mixture was allowed to reflux for 12 h. After completion of the reaction, the solvent was evaporated under vacuum and the crude product was washed with little amounts of pure water, methanol and toluene to get colourless crystals in 63% yield. M. p.: 461-463 K. ^1^H NMR (500 MHz, DMSO-d6): δ 8.044 (s, 1 H, imidazole H), 7.782–7.756 (m, 1 H, Ar H), 7.639–7.595 (m, 2 H, ArH), 7.295–7.263 (m, 1 H, Ar H), 5.397–5.334 (m, 1 H, CH), 4.650–4.421 (m, 2 H, CH_2_), 2.320 (s, 3 H, CH_3_), 1.392–1.379 (d, J=6.5 Hz, 3 H, CH_3_). ^13^C NMR (125 MHz, DMSO-d6): δ 167.93 (C—O), 167.60 (C—O), 152.08 (C—N), 139.02 (C—NO_2_), 133.65 (imidazole CH), 132.81, 132.03, 131.72, 131.52, 129.57, 127.83 (aromatic C), 70.70 (O—CH), 49.85 (N—CH_2_), 17.09 and 14.40 (CH_3_).

### S3. Refinement

The hydroxyl H atom was located in a difference Fourier map and refined freely. All other H atoms were placed in calculated positions and refined using a riding model approximation, with C—H = 0.93–0.98 Å and with *U*_iso_(H) = 1.2*U*_eq_(C) or 1.5*U*_eq_(C) for methyl H atoms.

### Crystal data

**Table:**

C_15_H_15_N_3_O_6_	*D*_x_ = 1.439 Mg m^−^^3^
*M**_r_* = 333.30	Melting point = 461–463 K
Monoclinic, *P*2_1_/*c*	Mo *K*α radiation, λ = 0.71073 Å
*a* = 11.189 (3) Å	Cell parameters from 9799 reflections
*b* = 6.9489 (17) Å	θ = 3.1–28.3°
*c* = 19.979 (5) Å	µ = 0.11 mm^−^^1^
β = 98.056 (10)°	*T* = 296 K
*V* = 1538.0 (6) Å^3^	Block, colourless
*Z* = 4	0.50 × 0.50 × 0.38 mm
*F*(000) = 696	

### Data collection

**Table:**

Bruker SMART APEX CCD area-detector diffractometer	2865 independent reflections
Radiation source: fine-focus sealed tube	2515 reflections with *I* > 2σ(*I*)
Graphite monochromator	*R*_int_ = 0.082
φ and ω scans	θ_max_ = 25.5°, θ_min_ = 3.1°
Absorption correction: multi-scan (*SADABS*; Bruker, 2000)	*h* = −13→13
*T*_min_ = 0.946, *T*_max_ = 0.958	*k* = −8→8
20047 measured reflections	*l* = −24→24

### Refinement

**Table:**

Refinement on *F*^2^	Secondary atom site location: difference Fourier map
Least-squares matrix: full	Hydrogen site location: mixed
*R*[*F*^2^ > 2σ(*F*^2^)] = 0.047	H atoms treated by a mixture of independent and constrained refinement
*wR*(*F*^2^) = 0.119	*w* = 1/[σ^2^(*F*_o_^2^) + (0.0414*P*)^2^ + 0.8462*P*] where *P* = (*F*_o_^2^ + 2*F*_c_^2^)/3
*S* = 1.07	(Δ/σ)_max_ < 0.001
2865 reflections	Δρ_max_ = 0.31 e Å^−^^3^
222 parameters	Δρ_min_ = −0.22 e Å^−^^3^
0 restraints	Extinction correction: *SHELXL2013* (Sheldrick, 2008), Fc^*^=kFc[1+0.001xFc^2^λ^3^/sin(2θ)]^-1/4^
Primary atom site location: structure-invariant direct methods	Extinction coefficient: 0.0086 (17)

### Special details

**Table:**

Geometry. All e.s.d.'s (except the e.s.d. in the dihedral angle between two l.s. planes) are estimated using the full covariance matrix. The cell e.s.d.'s are taken into account individually in the estimation of e.s.d.'s in distances, angles and torsion angles; correlations between e.s.d.'s in cell parameters are only used when they are defined by crystal symmetry. An approximate (isotropic) treatment of cell e.s.d.'s is used for estimating e.s.d.'s involving l.s. planes.
Refinement. Refinement on *F*^2^ for ALL reflections except those flagged by the user for potential systematic errors. Weighted *R*-factors *wR* and all goodnesses of fit *S* are based on *F*^2^, conventional *R*-factors *R* are based on *F*, with *F* set to zero for negative *F*^2^. The observed criterion of *F*^2^ > σ(*F*^2^) is used only for calculating -*R*-factor-obs *etc*. and is not relevant to the choice of reflections for refinement. *R*-factors based on *F*^2^ are statistically about twice as large as those based on *F*, and *R*-factors based on ALL data will be even larger.

### Fractional atomic coordinates and isotropic or equivalent isotropic displacement parameters (Å^2^)

**Table:**

	*x*	*y*	*z*	*U*_iso_*/*U*_eq_	
O1	0.06727 (13)	0.4222 (2)	0.34680 (7)	0.0516 (4)	
O2	0.23305 (12)	0.4311 (2)	0.29641 (7)	0.0545 (4)	
O3	0.28516 (14)	0.5351 (2)	0.15189 (11)	0.0768 (6)	
O4	0.26149 (10)	0.22096 (17)	0.17394 (6)	0.0375 (3)	
O5	0.58254 (14)	0.2723 (3)	0.08232 (9)	0.0720 (5)	
O6	0.57286 (14)	0.3388 (2)	−0.02413 (9)	0.0636 (5)	
N1	0.23187 (14)	0.1045 (2)	−0.03904 (8)	0.0441 (4)	
N2	0.35718 (12)	0.0837 (2)	0.05685 (7)	0.0329 (3)	
N3	0.52989 (14)	0.2715 (2)	0.02452 (9)	0.0469 (4)	
C1	0.12441 (16)	0.4255 (3)	0.29324 (9)	0.0382 (4)	
C2	0.04179 (15)	0.4222 (2)	0.22764 (9)	0.0346 (4)	
C3	−0.08314 (16)	0.4269 (3)	0.22600 (10)	0.0429 (4)	
H3A	−0.1161	0.4322	0.2662	0.051*	
C4	−0.15808 (17)	0.4237 (3)	0.16484 (11)	0.0501 (5)	
H4A	−0.2414	0.4246	0.1640	0.060*	
C5	−0.10993 (18)	0.4191 (3)	0.10507 (10)	0.0514 (5)	
H5A	−0.1608	0.4188	0.0640	0.062*	
C6	0.01374 (17)	0.4148 (3)	0.10593 (10)	0.0447 (5)	
H6A	0.0458	0.4120	0.0654	0.054*	
C7	0.09033 (15)	0.4146 (2)	0.16701 (9)	0.0349 (4)	
C8	0.22351 (16)	0.4028 (3)	0.16476 (9)	0.0386 (4)	
C9	0.39058 (15)	0.1846 (3)	0.17704 (9)	0.0382 (4)	
H9A	0.4320	0.3015	0.1653	0.046*	
C10	0.40426 (15)	0.0266 (3)	0.12629 (8)	0.0356 (4)	
H10A	0.4890	−0.0063	0.1286	0.043*	
H10B	0.3616	−0.0872	0.1382	0.043*	
C11	0.24736 (15)	0.0342 (3)	0.02342 (9)	0.0353 (4)	
C12	0.33437 (17)	0.2007 (3)	−0.04698 (9)	0.0447 (5)	
H12A	0.3487	0.2640	−0.0861	0.054*	
C13	0.41269 (15)	0.1901 (3)	0.01123 (9)	0.0364 (4)	
C14	0.4392 (2)	0.1213 (4)	0.24794 (10)	0.0598 (6)	
H14A	0.4299	0.2239	0.2790	0.090*	
H14B	0.5231	0.0895	0.2502	0.090*	
H14C	0.3953	0.0104	0.2597	0.090*	
C15	0.15837 (17)	−0.0872 (3)	0.05232 (10)	0.0472 (5)	
H15A	0.0877	−0.1030	0.0195	0.071*	
H15B	0.1366	−0.0263	0.0920	0.071*	
H15C	0.1932	−0.2110	0.0641	0.071*	
H1D	0.122 (3)	0.417 (4)	0.3880 (16)	0.086 (9)*	

### Atomic displacement parameters (Å^2^)

**Table:**

	*U*^11^	*U*^22^	*U*^33^	*U*^12^	*U*^13^	*U*^23^
O1	0.0439 (7)	0.0761 (10)	0.0338 (7)	0.0032 (7)	0.0023 (6)	−0.0029 (7)
O2	0.0375 (8)	0.0856 (11)	0.0385 (7)	0.0068 (7)	−0.0017 (5)	−0.0163 (7)
O3	0.0471 (9)	0.0618 (10)	0.1214 (16)	0.0013 (8)	0.0111 (9)	0.0439 (10)
O4	0.0314 (6)	0.0397 (7)	0.0410 (7)	0.0046 (5)	0.0036 (5)	−0.0054 (5)
O5	0.0431 (8)	0.0934 (13)	0.0748 (11)	−0.0162 (8)	−0.0079 (8)	0.0043 (10)
O6	0.0605 (9)	0.0540 (9)	0.0847 (12)	−0.0030 (7)	0.0398 (9)	−0.0002 (8)
N1	0.0427 (9)	0.0556 (10)	0.0325 (8)	0.0053 (7)	0.0001 (6)	0.0022 (7)
N2	0.0314 (7)	0.0358 (7)	0.0310 (7)	0.0046 (6)	0.0019 (5)	−0.0011 (6)
N3	0.0384 (9)	0.0403 (9)	0.0641 (11)	0.0026 (7)	0.0148 (8)	−0.0033 (8)
C1	0.0386 (10)	0.0386 (9)	0.0361 (9)	0.0061 (7)	0.0011 (7)	−0.0065 (7)
C2	0.0361 (9)	0.0308 (8)	0.0354 (9)	0.0042 (7)	−0.0001 (7)	−0.0030 (7)
C3	0.0366 (9)	0.0469 (11)	0.0448 (10)	0.0057 (8)	0.0047 (8)	−0.0063 (8)
C4	0.0311 (9)	0.0573 (12)	0.0591 (12)	0.0067 (9)	−0.0037 (8)	−0.0077 (10)
C5	0.0462 (11)	0.0580 (12)	0.0445 (11)	0.0091 (9)	−0.0133 (8)	−0.0067 (9)
C6	0.0462 (11)	0.0503 (11)	0.0356 (9)	0.0089 (9)	−0.0011 (8)	−0.0017 (8)
C7	0.0356 (9)	0.0305 (8)	0.0368 (9)	0.0043 (7)	−0.0014 (7)	−0.0005 (7)
C8	0.0390 (10)	0.0418 (10)	0.0338 (9)	0.0023 (8)	0.0013 (7)	0.0070 (7)
C9	0.0296 (9)	0.0463 (10)	0.0367 (9)	0.0053 (7)	−0.0021 (7)	−0.0069 (8)
C10	0.0334 (9)	0.0404 (9)	0.0312 (9)	0.0086 (7)	−0.0021 (7)	0.0013 (7)
C11	0.0323 (8)	0.0395 (9)	0.0331 (9)	0.0058 (7)	0.0007 (7)	−0.0038 (7)
C12	0.0480 (11)	0.0508 (11)	0.0365 (10)	0.0058 (9)	0.0102 (8)	0.0077 (8)
C13	0.0343 (9)	0.0355 (9)	0.0404 (9)	0.0035 (7)	0.0093 (7)	−0.0001 (7)
C14	0.0537 (12)	0.0858 (17)	0.0354 (10)	0.0165 (12)	−0.0089 (9)	−0.0103 (10)
C15	0.0399 (10)	0.0554 (12)	0.0451 (10)	−0.0058 (9)	0.0013 (8)	0.0002 (9)

### Geometric parameters (Å, º)

**Table:**

O1—C1	1.321 (2)	C4—H4A	0.9300
O1—H1D	0.96 (3)	C5—C6	1.382 (3)
O2—C1	1.209 (2)	C5—H5A	0.9300
O3—C8	1.199 (2)	C6—C7	1.389 (2)
O4—C8	1.337 (2)	C6—H6A	0.9300
O4—C9	1.459 (2)	C7—C8	1.499 (2)
O5—N3	1.221 (2)	C9—C14	1.510 (3)
O6—N3	1.235 (2)	C9—C10	1.517 (2)
N1—C11	1.329 (2)	C9—H9A	0.9800
N1—C12	1.356 (3)	C10—H10A	0.9700
N2—C11	1.358 (2)	C10—H10B	0.9700
N2—C13	1.386 (2)	C11—C15	1.482 (3)
N2—C10	1.468 (2)	C12—C13	1.356 (3)
N3—C13	1.418 (2)	C12—H12A	0.9300
C1—C2	1.494 (2)	C14—H14A	0.9600
C2—C3	1.394 (2)	C14—H14B	0.9600
C2—C7	1.396 (3)	C14—H14C	0.9600
C3—C4	1.382 (3)	C15—H15A	0.9600
C3—H3A	0.9300	C15—H15B	0.9600
C4—C5	1.377 (3)	C15—H15C	0.9600
			
C1—O1—H1D	111.9 (17)	O4—C9—C14	108.32 (15)
C8—O4—C9	117.58 (14)	O4—C9—C10	106.80 (13)
C11—N1—C12	106.87 (15)	C14—C9—C10	111.08 (16)
C11—N2—C13	105.64 (14)	O4—C9—H9A	110.2
C11—N2—C10	125.19 (15)	C14—C9—H9A	110.2
C13—N2—C10	129.14 (14)	C10—C9—H9A	110.2
O5—N3—O6	123.59 (18)	N2—C10—C9	112.30 (14)
O5—N3—C13	119.33 (17)	N2—C10—H10A	109.1
O6—N3—C13	117.08 (18)	C9—C10—H10A	109.1
O2—C1—O1	123.72 (16)	N2—C10—H10B	109.1
O2—C1—C2	122.70 (16)	C9—C10—H10B	109.1
O1—C1—C2	113.58 (15)	H10A—C10—H10B	107.9
C3—C2—C7	119.43 (16)	N1—C11—N2	111.01 (16)
C3—C2—C1	121.00 (16)	N1—C11—C15	124.67 (16)
C7—C2—C1	119.57 (15)	N2—C11—C15	124.30 (16)
C4—C3—C2	120.16 (18)	N1—C12—C13	109.17 (16)
C4—C3—H3A	119.9	N1—C12—H12A	125.4
C2—C3—H3A	119.9	C13—C12—H12A	125.4
C5—C4—C3	120.29 (18)	C12—C13—N2	107.31 (15)
C5—C4—H4A	119.9	C12—C13—N3	127.45 (17)
C3—C4—H4A	119.9	N2—C13—N3	125.24 (16)
C4—C5—C6	120.17 (18)	C9—C14—H14A	109.5
C4—C5—H5A	119.9	C9—C14—H14B	109.5
C6—C5—H5A	119.9	H14A—C14—H14B	109.5
C5—C6—C7	120.27 (18)	C9—C14—H14C	109.5
C5—C6—H6A	119.9	H14A—C14—H14C	109.5
C7—C6—H6A	119.9	H14B—C14—H14C	109.5
C6—C7—C2	119.67 (16)	C11—C15—H15A	109.5
C6—C7—C8	117.84 (16)	C11—C15—H15B	109.5
C2—C7—C8	122.49 (15)	H15A—C15—H15B	109.5
O3—C8—O4	124.94 (17)	C11—C15—H15C	109.5
O3—C8—C7	124.65 (17)	H15A—C15—H15C	109.5
O4—C8—C7	110.22 (15)	H15B—C15—H15C	109.5
			
O2—C1—C2—C3	−176.28 (18)	C8—O4—C9—C10	−127.65 (16)
O1—C1—C2—C3	3.6 (2)	C11—N2—C10—C9	−99.49 (19)
O2—C1—C2—C7	3.6 (3)	C13—N2—C10—C9	82.8 (2)
O1—C1—C2—C7	−176.53 (16)	O4—C9—C10—N2	61.80 (19)
C7—C2—C3—C4	0.1 (3)	C14—C9—C10—N2	179.71 (16)
C1—C2—C3—C4	−179.99 (17)	C12—N1—C11—N2	0.6 (2)
C2—C3—C4—C5	−1.1 (3)	C12—N1—C11—C15	−177.69 (17)
C3—C4—C5—C6	0.9 (3)	C13—N2—C11—N1	−0.60 (19)
C4—C5—C6—C7	0.2 (3)	C10—N2—C11—N1	−178.73 (15)
C5—C6—C7—C2	−1.1 (3)	C13—N2—C11—C15	177.73 (17)
C5—C6—C7—C8	177.87 (18)	C10—N2—C11—C15	−0.4 (3)
C3—C2—C7—C6	1.0 (3)	C11—N1—C12—C13	−0.4 (2)
C1—C2—C7—C6	−178.91 (16)	N1—C12—C13—N2	0.0 (2)
C3—C2—C7—C8	−177.98 (16)	N1—C12—C13—N3	−179.84 (17)
C1—C2—C7—C8	2.1 (3)	C11—N2—C13—C12	0.33 (19)
C9—O4—C8—O3	8.2 (3)	C10—N2—C13—C12	178.35 (16)
C9—O4—C8—C7	−176.63 (13)	C11—N2—C13—N3	−179.79 (16)
C6—C7—C8—O3	77.9 (3)	C10—N2—C13—N3	−1.8 (3)
C2—C7—C8—O3	−103.1 (2)	O5—N3—C13—C12	169.8 (2)
C6—C7—C8—O4	−97.27 (19)	O6—N3—C13—C12	−10.0 (3)
C2—C7—C8—O4	81.7 (2)	O5—N3—C13—N2	−10.1 (3)
C8—O4—C9—C14	112.64 (19)	O6—N3—C13—N2	170.11 (16)

### Hydrogen-bond geometry (Å, º)

**Table:**

*D*—H···*A*	*D*—H	H···*A*	*D*···*A*	*D*—H···*A*
C9—H9*A*···O5	0.98	2.53	3.115 (3)	118
O1—H1*D*···N1^i^	0.96 (3)	1.78 (3)	2.730 (2)	175 (3)

Symmetry code: (i) *x*, −*y*+1/2, *z*+1/2.
