# Supplementary figures and images for: Crystal structure of 2-{[1-(2-methyl-5-nitro-1H-imidazol-1-yl)propan-2-yl­oxy]carbon­yl}benzoic acid
Source: Acta Crystallogr Sect E Struct Rep Online. 2014 Nov 5;70(Pt 12):o1237–8. doi: 10.1107/S1600536814023927 (PMC4257427; doi:10.1107/S1600536814023927)

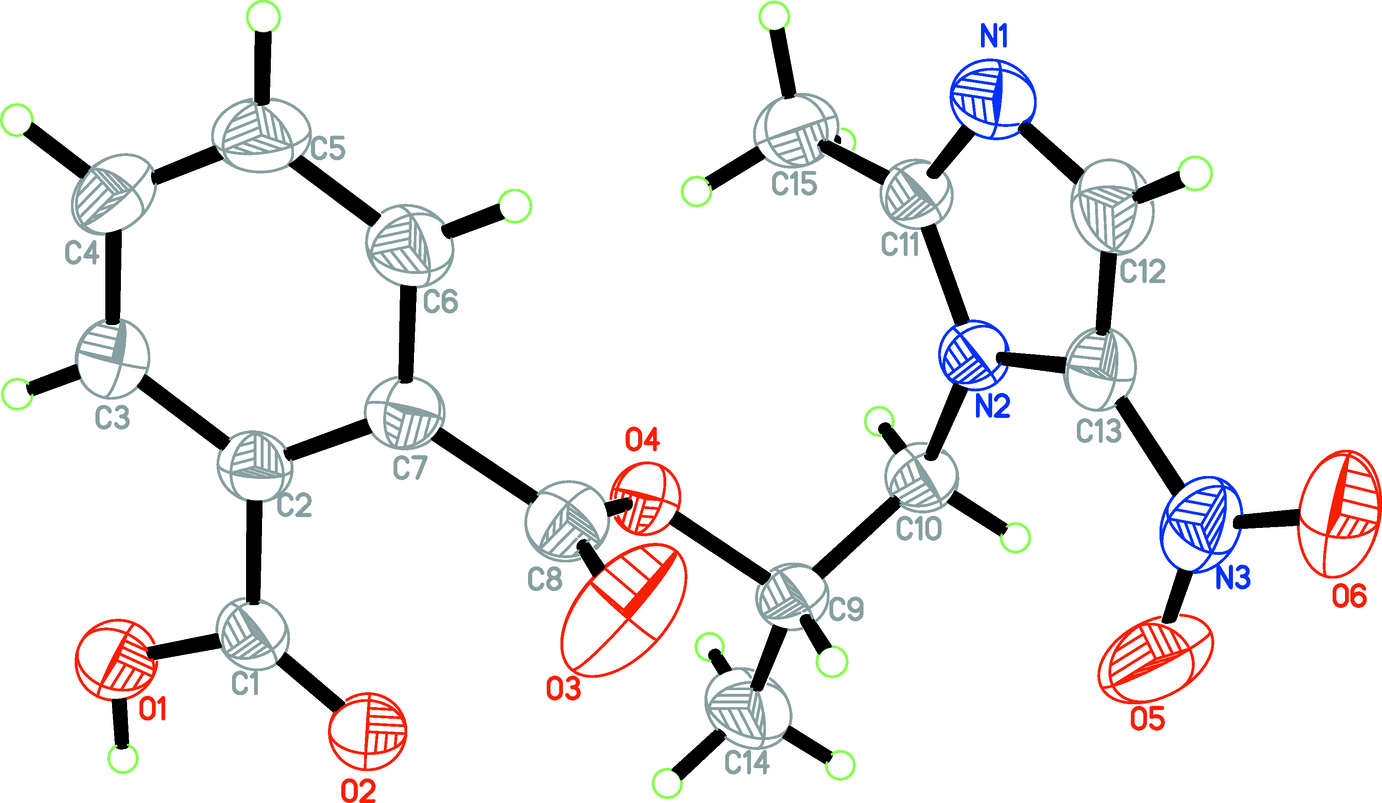

Supplement: Supplementary file 4 [file e-70-o1237-fig1.tif]

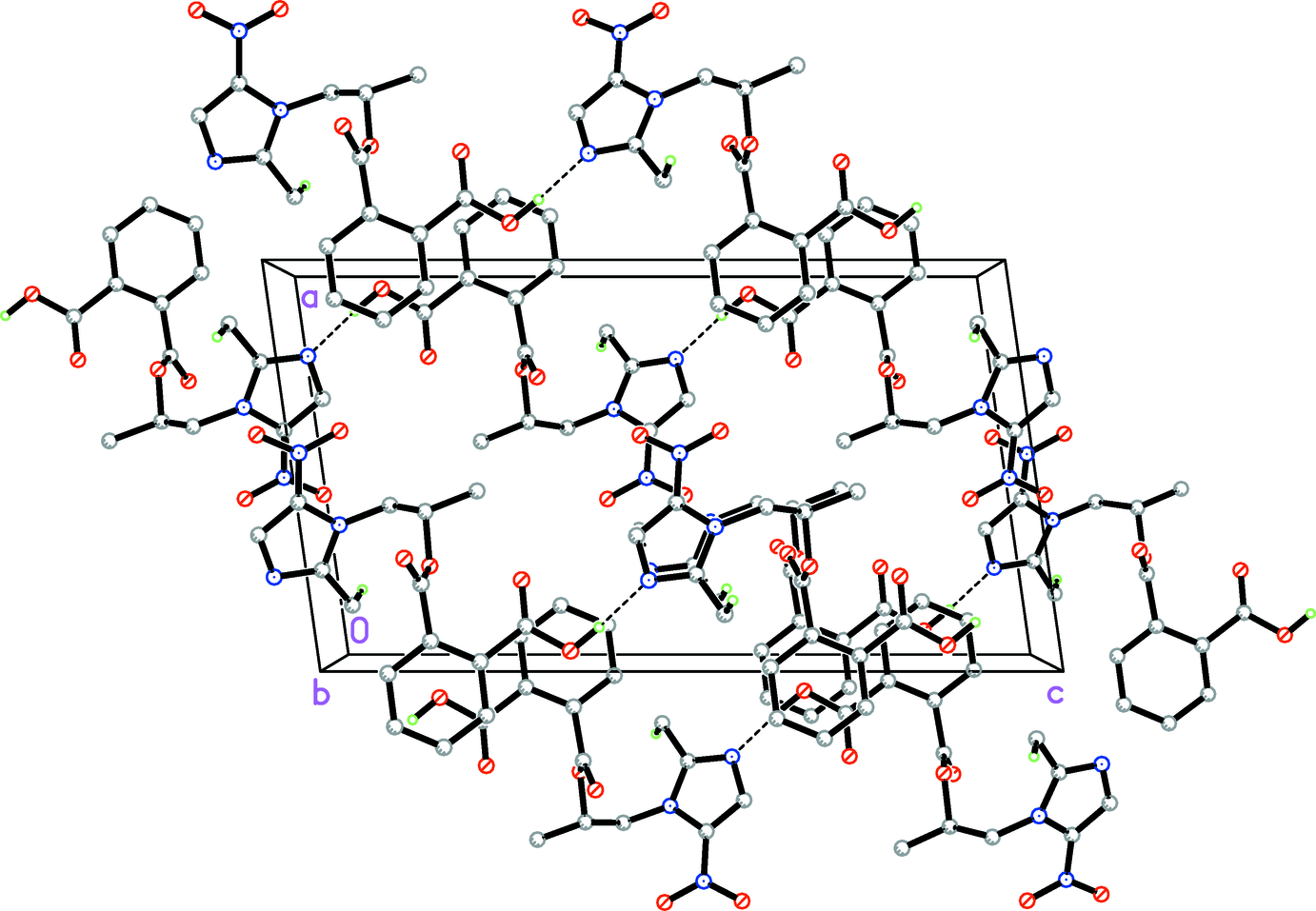

Supplement: Supplementary file 5 [file e-70-o1237-fig2.tif]
